# Limited diagnostic utility of albumin-corrected calcium in the intensive care unit: A prospective comparison with ionised calcium

**DOI:** 10.1371/journal.pone.0354233

**Published:** 2026-07-21

**Authors:** Erdoğan Özdemir, Turgay Yılmaz, Deccane Düzenci

**Affiliations:** 1 Department of Internal Medicine, Fethi Sekin City Hospital, Elazığ, Türkiye; 2 Department of Intensive Care Unit, Fethi Sekin City Hospital, Elazığ, Türkiye; University of Health Sciences, Beyhekim Training and Research Hospital, TÜRKIYE

## Abstract

**Background:**

Albumin-adjusted calcium is widely used despite uncertainty about its accuracy in critically ill adults. We prospectively evaluated the diagnostic performance of total calcium and of multiple published albumin-correction formulae against ionised calcium, the physiological reference standard, in an intensive care unit (ICU) cohort.

**Methods:**

In this prospective observational cohort study, ionised calcium (iCa), total calcium, albumin, venous pH, creatinine, estimated glomerular filtration rate (eGFR), C-reactive protein (CRP), procalcitonin, magnesium, phosphate and vitamin D were measured simultaneously. The primary outcome was the diagnostic accuracy (area under the ROC curve [AUC]; sensitivity/specificity) of total calcium and of corrected calcium for detecting hypocalcaemia, defined a priori by the reference standard as iCa < 1.12 mmol/L; the pre-specified contrast was the difference between the two correlated AUCs. Twelve published measures were compared (total calcium; Payne; simplified Payne; Orrell; Berry; James; Jain; Thode; and the Pekar and Antonio estimators). Agreement, formal subgroup interaction tests, an explanatory multivariable model with collinearity diagnostics, and an exploratory internally-validated ICU equation were assessed.

**Results:**

In total, 250 patients were enrolled (median age 70 years; 147 men, 58.8%). Hypocalcaemia prevalence was 58.0% (n = 145). Payne-corrected calcium correctly identified only 15.2% of truly hypocalcaemic patients (Cohen κ = 0.08), versus 62.8% for total calcium (κ = 0.40). No simple albumin-correction formula significantly outperformed unadjusted total calcium (AUC 0.69–0.75; all DeLong p > 0.05 vs total calcium AUC 0.74). Only the four-variable Pekar estimator achieved a higher AUC (0.78; p = 0.005). In the explanatory model, only total calcium, pH, and sex independently predicted iCa (all variance inflation factors < 1.8); albumin, phosphate and vitamin D were not significant. An exploratory ICU-specific equation reached an optimism-corrected R² of only 0.17.

**Conclusions:**

Albumin-corrected calcium, including all established formulae tested, provides no diagnostic benefit over total calcium in the ICU and frequently masks true hypocalcaemia. Direct ionised-calcium measurement should be prioritised when accurate calcium status affects management. These are diagnostic-accuracy findings; their clinical-outcome implications require outcome-anchored study.

## Introduction

Calcium homeostasis is essential for neuromuscular excitability, cardiac contractility, signal transduction, coagulation and skeletal mineralisation. Only ~45% of circulating calcium exists in the physiologically active ionised fraction (iCa), the remainder being bound to albumin or complexed with anions [1,2]. Ionised calcium is the only biologically valid fraction but its measurement is highly vulnerable to pre-analytical conditions—immediate anaerobic processing, appropriate heparinisation and pH correction—so total calcium or the albumin-adjusted (Payne) calcium are widely substituted, particularly outside the ICU. The Payne formula was derived in 1973 from non-critically ill adults using the bromocresol green albumin method and may not generalise to contemporary ICU populations [3,4].

High-quality studies consistently show that albumin-adjusted calcium does not reliably reflect ionised calcium and frequently masks true hypocalcaemia [5–7]. However, this evidence is largely derived from retrospective datasets, non-simultaneous sampling, heterogeneous analysers and limited pre-analytical control—conditions known to compromise the validity of ionised-calcium measurement—and prospective studies that simultaneously benchmark multiple published correction formulae against a rigorously obtained reference standard, and that systematically examine biological modifiers, remain scarce.

Using prospectively collected, strictly simultaneous sampling with anaerobic transport, automated pH correction and single-analyser measurement, this study compared the diagnostic accuracy of total calcium and Payne-corrected calcium—and, additionally, of ten further published correction or estimation equations—for detecting hypocalcaemia in adult ICU patients, using simultaneously measured ionised calcium as the reference standard, and explored how performance varies across clinically relevant subgroups.

## Materials and methods

### Study design and ethics

This prospective observational cohort study was conducted between 18 December 2025 and 28 February 2026 in the general intensive care units of Elazığ Fethi Sekin City Hospital. The study was performed in accordance with the Declaration of Helsinki and approved by the institutional ethics committee (approval no. 2025/21–26). Written informed consent was obtained from all patients or their legally authorised representatives. No vulnerable populations were enrolled without appropriate proxy consent.

### Eligibility and patient selection

All adult patients admitted to the ICU during the study period were screened. Patients were included if aged ≥ 18 years with simultaneously measured serum total calcium, serum albumin and ionised calcium, plus same-day venous pH, creatinine, estimated glomerular filtration rate (eGFR), C-reactive protein (CRP), procalcitonin, magnesium and phosphate. Patients were excluded for: haemolysed/poor-quality samples; calcium supplementation on the sampling day; medications that independently perturb calcium homeostasis (loop/thiazide diuretics, bisphosphonates, calcimimetics, active vitamin D analogues, systemic corticosteroids, enzyme-inducing antiepileptics, fluoroquinolones); untreated parathyroid disease, parathyroidectomy or recent thyroidectomy; or conditions that markedly disrupt calcium balance (e.g., rhabdomyolysis, tumour lysis syndrome). These exclusions were pre-specified to isolate the analytical question—whether the albumin term of correction formulae recovers ionised calcium—by removing non-albumin-mediated calcium derangements.

**Fig 1:**
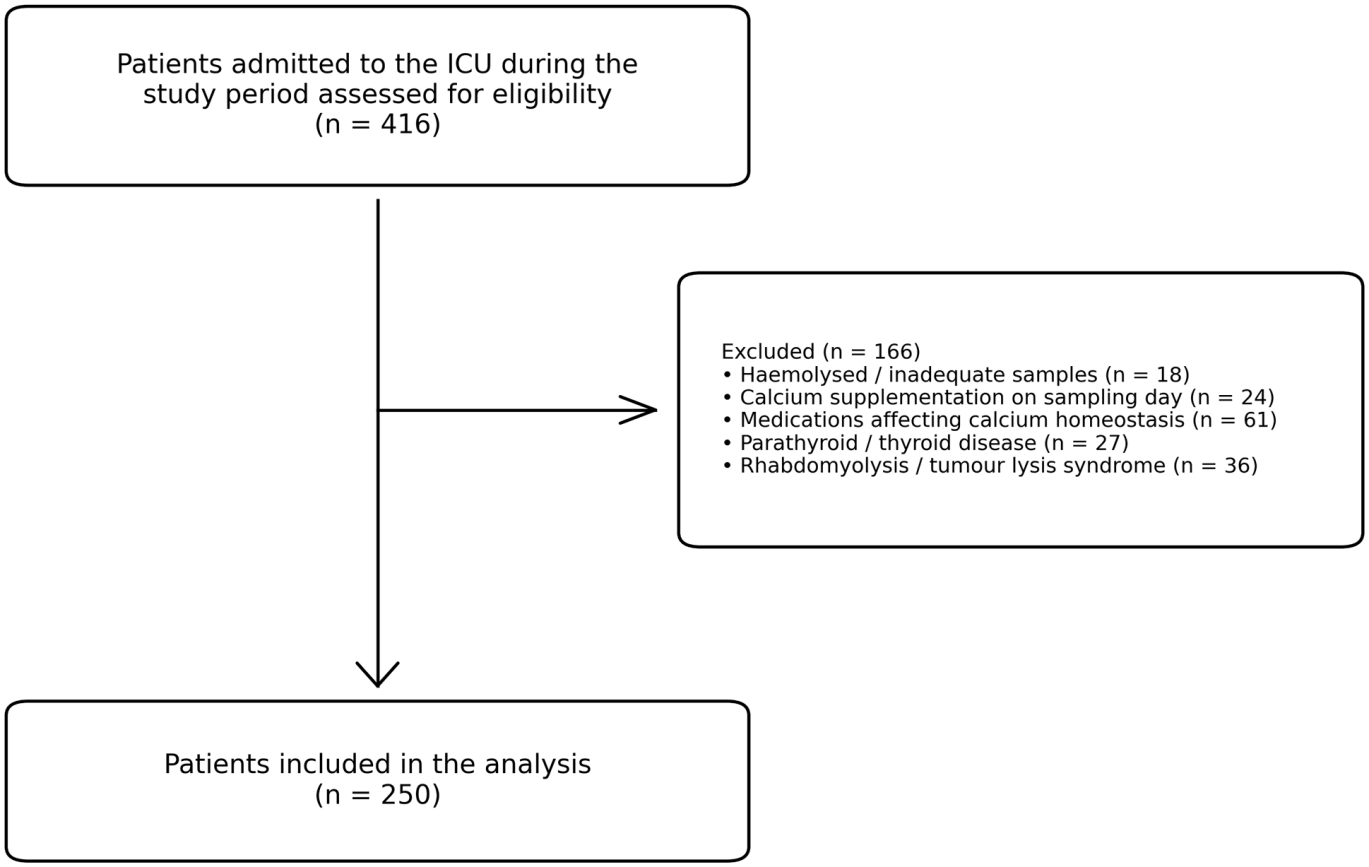
Study flow diagram. Of 416 ICU patients assessed for eligibility, 166 were excluded (haemolysed/inadequate samples, n = 18; calcium supplementation on the sampling day, n = 24; medications affecting calcium homeostasis, n = 61; parathyroid/thyroid disease, n = 27; rhabdomyolysis/tumour lysis syndrome, n = 36), leaving 250 patients in the analysis.

An a priori sample-size calculation for the secondary explanatory model (G*Power 3.1.9.7; linear multiple regression, ten predictors, medium effect, two-sided α = 0.05, power 80%) indicated a minimum of 200 patients; a target of 250 was set to additionally support the pre-specified exploratory subgroup analyses. Of 416 patients screened, 166 were excluded (haemolysed/inadequate samples, n = 18; calcium supplementation, n = 24; medications affecting calcium, n = 61; parathyroid/thyroid disease, n = 27; rhabdomyolysis/tumour lysis, n = 36); after applying the eligibility criteria, 250 patients were included in the analysis (Fig 1).

### Laboratory measurements

Ionised calcium was measured on a Radiometer ABL90 FLEX analyser; samples were collected in balanced-heparin syringes, transported anaerobically and analysed within 15 minutes with automated pH correction at 37 °C. Total calcium (Arsenazo III) and albumin (bromocresol green) were measured on a Beckman Coulter AU5800. Albumin-adjusted calcium was calculated by the Payne formula: corrected calcium (mg/dL) = total calcium + 0.8 × (4.0 − albumin [g/dL]) [4]. Ten additional published measures were computed for comparison: the simplified Payne, Orrell, Berry, James, Jain and Thode corrected-calcium formulae, and the Pekar and Antonio (A/B/C) estimators of ionised calcium, using each formula’s original published coefficients [4,6,8,9]; SI conversions used calcium (mmol/L) = mg/dL ÷ 4.008 and albumin (g/L) = g/dL × 10.

### Primary outcome and reference standard

Ionised calcium was the reference standard. Consistent with the Radiometer ABL90 FLEX reference interval and thresholds widely used in ICU calcium studies [3], hypocalcaemia was defined a priori as iCa < 1.12 mmol/L, normocalcaemia as 1.12–1.32 mmol/L and hypercalcaemia as > 1.32 mmol/L. The primary outcome was the diagnostic accuracy of total calcium and of Payne-corrected calcium for detecting hypocalcaemia, quantified by the AUC and by sensitivity/specificity at the conventional clinical threshold (8.5 mg/dL); the pre-specified comparative contrast was the difference between the two correlated AUCs.

### Statistical analysis

Analyses used IBM SPSS Statistics 29.0 and MedCalc 22.017. Continuous variables are summarised as mean ± standard deviation (SD) or median (interquartile range [IQR]), categorical variables as counts (percentages). Agreement was assessed by cross-classification, overall accuracy, misclassification rates and Cohen’s κ. Diagnostic performance used ROC analysis with AUC and bootstrap 95% confidence intervals; correlated AUCs were compared by the DeLong method. Because total/corrected calcium (mg/dL) and ionised calcium (mmol/L) are not the same measurand on a common scale, classification metrics against the clinical decision threshold were the primary analysis; Bland–Altman analysis was additionally performed with corrected/total calcium expressed on the ionised-calcium scale (per the convention of Byrnes et al. [10]) and natively for the Pekar/Antonio estimators. Subgroup analyses (albumin, pH, CRP, magnesium, phosphate, procalcitonin, eGFR, vitamin D, and major diagnostic groups) were pre-specified as exploratory; heterogeneity was tested formally with logistic models including a calcium-measure × subgroup interaction term, interpreted with a multiple-comparisons caveat. An explanatory multivariable linear model of ionised calcium was fitted with standardised coefficients and variance inflation factors (VIF); a sensitivity model excluding total calcium was also fitted. An exploratory ICU-specific equation was derived by linear regression and internally validated by bootstrap optimism correction (1000 resamples). For a diagnostic-accuracy objective comparing two correlated AUCs, the achieved precision (AUC 95% CI half-width) and the minimum detectable between-test AUC difference at 80% power (two-sided α = 0.05) were computed. p < 0.05 was considered significant.

## Results

Of 250 patients, 147 (58.8%) were male and 103 (41.2%) female. The commonest primary diagnoses were chronic obstructive pulmonary disease (COPD, n = 49, 19.6%), cerebrovascular disease (n = 43, 17.2%), pneumonia (n = 32, 12.8%), acute coronary syndrome (ACS, n = 23, 9.2%), heart failure (n = 16, 6.4%) and trauma (n = 14, 5.6%); other diagnoses accounted for the remainder. Median age was 70 years (IQR 58–80). Descriptive statistics are shown in Table 1.

**Table 1:** Patient characteristics (n = 250).

Variable	Mean	SD	Min	P25	Median	P75	Max
Age (years)	66.6	17.7	18	58	70	80	97
pH	7.37	0.12	6.96	7.33	7.40	7.45	7.69
Ionised calcium (mmol/L)	1.09	0.11	0.65	1.04	1.10	1.16	1.42
CRP (mg/L)	79.9	98.9	1	9	27	132	467
eGFR (mL/min/1.73m²)	69.3	40.8	2.0	33	68.5	95	184
Creatinine (mg/dL)	1.41	1.27	0.17	0.70	0.95	1.71	9.63
Albumin (g/dL)	3.07	0.74	0.34	2.60	3.10	3.60	4.60
Total calcium (mg/dL)	8.51	0.92	5.20	8.00	8.60	9.10	10.90
Magnesium (mg/dL)	2.01	0.38	0.38	1.80	1.97	2.23	3.50
Phosphate (mg/dL)	3.73	1.62	1.17	2.72	3.48	4.21	13.45
Corrected calcium (mg/dL)	9.25	0.74	5.70	8.88	9.26	9.68	11.92
Vitamin D (ng/mL)	17.96	16.36	1	10	15	22	170
Procalcitonin (ng/mL)	6.08	16.68	0.0	0.07	0.46	2.69	95.49

CRP, C-reactive protein; eGFR, estimated glomerular filtration rate; IQR, interquartile range; P25/P75, 25th/75th percentile; SD, standard deviation.

Based on ionised calcium, 145 patients (58.0%) were hypocalcaemic, 102 (40.8%) normocalcaemic and 3 (1.2%) hypercalcaemic (Table 2). Of the 145 truly hypocalcaemic patients, the Payne formula correctly identified only 22 (15.2%), misclassifying 123 (84.8%) as normocalcaemic; total calcium correctly identified 91 (62.8%), misclassifying 54 (37.2%) as normocalcaemic.

**Table 2:** Calcium status classification by each measure.

Calcium status	Ionised calcium (n)	Total calcium (n)	Corrected calcium (n)
Hypocalcaemia	145	113	28
Normocalcaemia	102	135	213
Hypercalcaemia	3	2	9

iCa thresholds: < 1.12 mmol/L (hypocalcaemia), 1.12–1.32 mmol/L (normocalcaemia), > 1.32 mmol/L (hypercalcaemia). Total/Payne threshold 8.5 mg/dL. iCa, ionised calcium.

Table 3 summarises the diagnostic performance of all twelve measures for detecting hypocalcaemia. No simple albumin-correction formula (Payne, simplified Payne, Orrell, Berry, James, Jain, Thode) significantly outperformed unadjusted total calcium (all DeLong p > 0.05). Only the Pekar estimator—a four-variable regression incorporating albumin, creatinine, pH and total calcium, i.e., not a simple albumin adjustment—achieved a higher AUC (0.78; p = 0.005). The albumin-based formulae achieved high specificity at the cost of very low sensitivity (e.g., Payne sensitivity 15.2%).

**Table 3:** Diagnostic performance for detecting hypocalcaemia.

Measure	AUC (95% CI)	Sens %	Spec %	κ	p vs total Ca
Total calcium	0.740 (0.678–0.801)	62.8	79.0	0.40	ref
Payne	0.712 (0.646–0.778)	15.2	94.3	0.08	0.356
Simplified Payne	0.711 (0.645–0.777)	15.2	94.3	0.08	0.344
Orrell	0.724 (0.660–0.788)	42.8	82.9	0.24	0.563
Berry	0.695 (0.627–0.762)	6.9	98.1	0.04	0.179
James	0.743 (0.681–0.804)	29.7	91.4	0.19	0.856
Jain	0.747 (0.686–0.808)	61.4	76.2	0.36	0.583
Thode	0.709 (0.643–0.775)	14.5	94.3	0.08	0.326
Pekar	0.782 (0.725–0.839)	31.0	96.2	0.24	0.005
Antonio A	0.740 (0.678–0.801)	21.4	97.1	0.16	1.000
Antonio B	0.724 (0.660–0.788)	22.1	93.3	0.14	0.123
Antonio C	0.747 (0.686–0.809)	19.3	98.1	0.15	0.347

Reference standard: iCa < 1.12 mmol/L. Sensitivity/specificity at the conventional clinical threshold (8.5 mg/dL for total/corrected calcium). p = DeLong comparison of AUC versus total calcium. AUC, area under the receiver operating characteristic curve; CI, confidence interval; iCa, ionised calcium; κ, Cohen’s kappa; Sens, sensitivity; Spec, specificity.

In the explanatory multivariable model (adjusted R² = 0.22), only total calcium (standardised β = +0.48, p < 0.001), pH (β = −0.24, p < 0.001), and sex (β = +0.13, p = 0.028) independently predicted ionised calcium; albumin (β = −0.11, p = 0.155), magnesium, phosphate (β = −0.12, p = 0.09), vitamin D (β = +0.03, p = 0.61), eGFR, CRP, procalcitonin and age were not significant (Table 4). All variance inflation factors were < 1.8 (total calcium VIF = 1.45). A sensitivity model excluding total calcium had an adjusted R² of only 0.06, with pH, magnesium and phosphate retaining significance.

**Table 4:** Multivariable model predicting ionised calcium.

Variable	Std β	95% CI	p	VIF
Total calcium	+0.48	0.35 to 0.62	<0.001	1.45
Albumin	−0.11	−0.25 to 0.04	0.155	1.78
pH	−0.24	−0.36 to −0.11	<0.001	1.36
Magnesium	+0.09	−0.02 to 0.21	0.121	1.15
Phosphate	−0.12	−0.25 to 0.02	0.089	1.54
eGFR	+0.02	−0.12 to +0.17	0.773	1.74
CRP	+0.01	−0.13 to 0.14	0.896	1.51
Procalcitonin	+0.03	−0.09 to 0.16	0.621	1.30
Age	−0.06	−0.18 to 0.06	0.336	1.23
Sex (male)	+0.13	0.01 to 0.25	0.028	1.10
Vitamin D	+0.03	−0.08 to 0.14	0.614	1.04

Standardised regression coefficients. CI, confidence interval; CRP, C-reactive protein; eGFR, estimated glomerular filtration rate; Std β, standardised regression coefficient; VIF, variance inflation factor.

**Fig 2:**
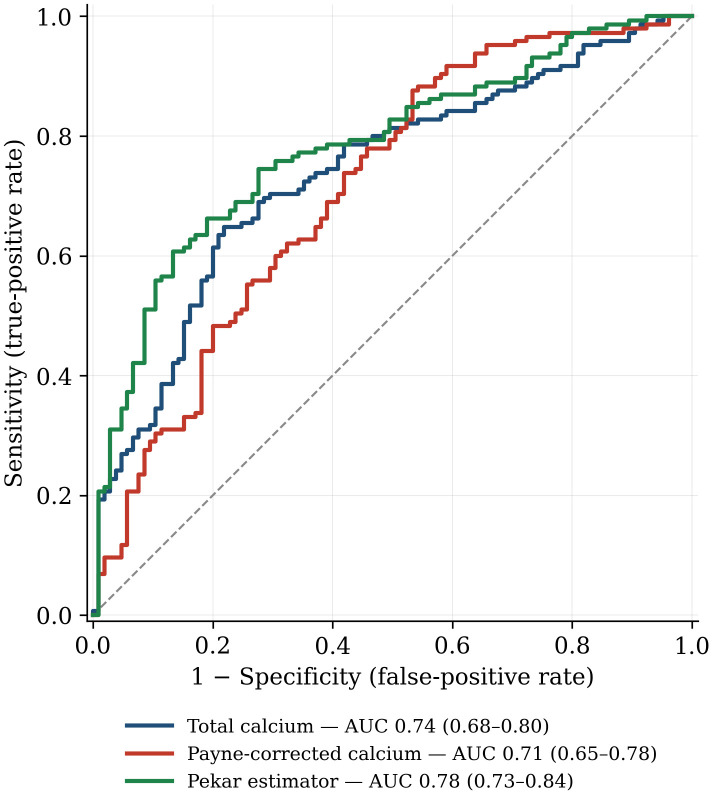
Receiver operating characteristic (ROC) curves for the detection of hypocalcaemia (ionised calcium < 1.12 mmol/L). Areas under the curve (95% CI) were 0.74 (0.68–0.80) for total calcium, 0.71 (0.65–0.78) for Payne-corrected calcium, and 0.78 (0.73–0.84) for the Pekar estimator. The diagonal dashed line denotes chance-level performance.

For the primary contrast, total-calcium AUC was 0.74 (95% CI 0.68–0.80) and Payne 0.71 (0.65–0.78), with no significant difference (DeLong p = 0.356) (Fig 2). With 145 events and 105 non-events the achieved 95% CI half-width for an AUC of ≈ 0.74 was ≈ 0.06; the cohort had 80% power to detect a between-test AUC difference of ≈ 0.08. The observed difference (0.03) is below this threshold.

Bland–Altman analysis showed wide 95% limits of agreement for every measure (approximately ±0.25 mmol/L on the ionised-calcium scale) with non-trivial systematic bias (e.g., Payne bias +0.06 mmol/L, limits −0.20 to +0.32), reinforcing the classification findings.

Exploratory subgroup analyses (Tables 5 and 6) are descriptive only. AUC values were numerically higher for both measures in marked hypoalbuminaemia (< 2.5 g/dL), markedly elevated CRP (> 100 mg/L) and alkalosis (pH > 7.45), and lower with normal/high albumin and moderate renal impairment. Formal interaction tests were nominally significant for albumin stratum and inflammatory markers (e.g., CRP > 100 mg/L, procalcitonin > 2 ng/mL). No between-measure difference reached significance within any diagnostic subgroup.

**Table 5:** Subgroup analysis: AUC by selected strata.

Subgroup	n	AUC total (95% CI)	AUC Payne (95% CI)	Interaction p
Albumin <2.5	57	0.841 (0.736–0.947)	0.848 (0.744–0.953)	0.048
Albumin 2.5–3.5	116	0.779 (0.692–0.867)	0.783 (0.694–0.873)	—
Albumin >3.5	77	0.586 (0.458–0.715)	0.562 (0.432–0.692)	<0.001
CRP > 100	78	0.824 (0.724–0.924)	0.878 (0.799–0.957)	0.035
Procalcitonin >2	73	0.800 (0.681–0.918)	0.873 (0.778–0.968)	0.034
pH > 7.45	52	0.841 (0.732–0.950)	0.825 (0.692–0.957)	0.090
eGFR < 30	55	0.776 (0.651–0.901)	0.873 (0.767–0.978)	0.303

Interaction p = logistic model with calcium-measure × subgroup interaction term; — = not computed. AUC, area under the receiver operating characteristic curve; CI, confidence interval; CRP, C-reactive protein; eGFR, estimated glomerular filtration rate.

**Table 6:** Subgroup analysis: AUC by primary diagnosis.

Diagnosis	n	AUC total (95% CI)	AUC Payne (95% CI)
COPD	49	0.689 (0.540–0.838)	0.491 (0.320–0.662)
Cerebrovascular disease	43	0.784 (0.642–0.925)	0.822 (0.691–0.954)
Pneumonia	32	0.749 (0.581–0.917)	0.787 (0.629–0.946)
ACS	23	0.830 (0.654–1.000)	0.716 (0.495–0.937)
Heart failure	16	0.615 (0.139–1.000)	0.487 (0.000–1.000)
Trauma	14	0.778 (0.508–1.000)	0.833 (0.597–1.000)

Categories with n < 10 not analysed. ACS, acute coronary syndrome; AUC, area under the receiver operating characteristic curve; CI, confidence interval; COPD, chronic obstructive pulmonary disease.

An exploratory single-centre ICU-specific equation (iCa [mmol/L] = 2.447 + 0.047 × total calcium − 0.014 × albumin − 0.228 × pH − 0.009 × phosphate) had an apparent R^2^ of 0.23; after bootstrap optimism correction, R^2^ was 0.17 and AUC for hypocalcaemia was 0.76.

## Discussion

In this prospective ICU cohort, Payne-corrected calcium misclassified the large majority of truly hypocalcaemic patients as normocalcaemic, whereas unadjusted total calcium performed modestly better but still failed in more than one third of cases. Critically, none of seven established albumin-correction formulae significantly outperformed total calcium, and an equation derived on these very data did not exceed it after internal validation. Neither total nor any albumin-corrected calcium is a dependable substitute for direct ionised-calcium measurement in critically ill adults.

These findings are consistent with, and extend, prior work. Earlier analyses [5–7,9,11–13] reported poor agreement between adjusted and ionised calcium and frequent masking of hypocalcaemia, but were largely retrospective, used non-simultaneous sampling and heterogeneous analysers, and rarely benchmarked multiple formulae against a rigorously obtained reference standard. By using strictly simultaneous sampling, anaerobic transport, automated pH correction and single-analyser measurement, and by comparing twelve published measures head-to-head, the present study provides confirmatory evidence under the methodological conditions prior studies generally lacked.

The persistent failure of albumin-based correction is biologically expected. The Payne formula assumes a fixed albumin–calcium binding factor anchored to an albumin of 4.0 g/dL and was derived in non-critically ill adults with the bromocresol green method [4], which overestimates albumin in the presence of acute-phase proteins [14]; in critical illness, true albumin is reduced by inflammation, capillary leak and malnutrition while measured albumin may be falsely elevated [15–17]. Applying a historical fixed factor to this milieu predisposes to systematic misclassification. Our multivariable analysis supports this: ionised calcium was independently predicted by total calcium, pH, and sex—pH reflecting the well-established pH-dependence of albumin calcium binding—while albumin contributed no independent information, consistent with hypoalbuminaemia in critical illness reflecting systemic processes rather than altered calcium-binding dynamics [18,19]. That the model explained only ~22% of ionised-calcium variance, and that a bespoke ICU equation reached only ~17% after internal validation, indicates that ionised calcium cannot be recovered from routine biochemistry and must be measured directly.

Exploratory subgroup analyses suggested numerically higher discrimination in marked hypoalbuminaemia, intense inflammation and alkalosis, with nominally significant interactions for albumin and inflammatory strata; however, wide confidence intervals, uncorrected multiplicity and the cohort’s limited power to detect small AUC differences (minimum detectable ≈ 0.08) preclude firm conclusions. These signals are best regarded as hypotheses for adequately powered, multicentre evaluation, potentially using data-driven subphenotyping (e.g., latent-class analysis) to identify stable calcium-handling clusters [20].

### Future directions

Because this is a diagnostic-accuracy study against a biochemical reference, it cannot establish whether misclassification by corrected calcium changes management or patient-important outcomes. An outcome-anchored design—ideally a target-trial-emulation framework comparing management guided by corrected versus ionised calcium and estimating effects on mortality, arrhythmia and length of stay—would be required to quantify clinical impact and cost-effectiveness [21,22].

### Strengths and limitations

Strengths include the prospective design with standardised, rapid, anaerobic ionised-calcium measurement and automated pH correction; an unselected ICU population with frequent hypoalbuminaemia and acid–base disturbance; head-to-head comparison of twelve published measures; and formal collinearity and interaction diagnostics. Limitations include the single-centre design and the high exclusion rate (39.9%), which yields a population enriched for albumin/acid–base-mediated calcium disturbance and under-representing pharmacologically complex patients; because excluded patients’ laboratory data were not retained, included-versus-excluded comparison and quantification of selection bias are not possible. Albumin was measured only by bromocresol green, so method-specific bias cannot be excluded. Clinical outcomes and treatment decisions were not captured, so the clinical consequences of misclassification cannot be inferred. The explanatory model left most ionised-calcium variance unexplained, and subgroup analyses were underpowered with wide confidence intervals. Larger, multicentre, outcome-anchored studies are warranted.

## Conclusions

In critically ill adults, albumin-corrected calcium—using the Payne formula or any of the other established formulae evaluated—provides no diagnostic advantage over unadjusted total calcium and frequently masks true hypocalcaemia. Total calcium performs modestly but remains inferior to direct ionised-calcium measurement, which should be prioritised whenever accurate calcium status is clinically important. These conclusions are based on diagnostic-accuracy data; their implications for clinical decisions and outcomes require dedicated outcome-anchored investigation.
